# Clinicopathological profile and survival in children with parameningeal rhabdomyosarcoma in resource-limited settings: A single-center experience from Uganda

**DOI:** 10.1371/journal.pone.0334140

**Published:** 2025-10-09

**Authors:** Richard Nyeko, Fadhil Geriga, Racheal Angom, Joyce Balagadde Kambugu, Jaques van Heerden

**Affiliations:** 1 Department of Pediatrics and Child Health, Lira University, Lira, Uganda; 2 Paediatric Oncology Division, Uganda Cancer Institute, Kampala, Uganda; 3 Department of Pediatric Hemato-Oncology, Antwerp University Hospital, University of Antwerp, Antwerp, Belgium; All India Institute of Medical Sciences, INDIA

## Abstract

**Background:**

Parameningeal rhabdomyosarcoma (PM-RMS) represents a diagnostic and therapeutic challenge, especially in low- and middle-income countries (LMICs), given its location and its propensity for local and intracranial extension. This study determined the clinical and pathological profile and survival in children with PM-RMS treated at a single tertiary cancer treatment facility in Uganda.

**Methods:**

This was a retrospective study involving a review of records of children and adolescents aged below 18 years with rhabdomyosarcoma treated at the Uganda Cancer Institute (UCI) between January 2016 and December 2020. Kaplan-Meier survival analysis and Cox’s proportional hazards model were used for five-year survival analysis.

**Results:**

We identified 32 PM-RMS cases with a median age of 4.8 years (range 1–15 years). The most common tumor sites were the infratemporal region (25.0%), middle ear (21.8%), and nasopharynx (18.8%). Most patients (90.6%) were IRS group III, and 34.4% had regional lymph node involvement. Embryonal and alveolar histologies represented 46.9% and 21.8%, respectively, with 31.3% unclassified. Nearly all patients (90.6%) received chemotherapy, but only 43.8% underwent radiotherapy for local control. One- and five-year overall survival rates were 65% and 12%, respectively. Regional nodal involvement and receipt of local control were the significant predictors of survival (adjusted HR 4.61 and 6.07, respectively).

**Conclusion:**

Our study demonstrates a low survival rate among children with PM-RMS among the patient cohort, and treatment abandonment remains high. Regional nodal involvement and local control significantly predicted survival.

## Background

Parameningeal rhabdomyosarcoma (PM-RMS) is a sub-classification of rhabdomyosarcoma (RMS) with a distinct pathophysiology and poor prognosis [1,2]. PM-RMS encompasses tumors arising from the parameningeal region, including the nasopharynx, nasal cavity, parapharyngeal space, paranasal sinuses, infratemporal and pterygopalatine fossa, middle ear, and mastoid, or from outside this region but with parameningeal/intracranial extension [2,3]. Twenty percent of RMS occurs in the parameningeal area [3].

Treatment of PM-RMS, just as for RMS in general, relies on the use of a multimodal therapeutic approach involving a combination of multi-agent therapy combined with effective local control using surgery (primary or secondary) and radiotherapy (RT) [4–6]. Local control of the primary tumor, which can arise at many distinct anatomic sites, as well as control of disseminated disease, remains the hallmark of curative therapy in RMS in general [6].

However, parameningeal sites of RMS have been shown to portend an unfavorable prognosis compared to other sites of origin [1,3,7]. The complex anatomy and proximity to critical anatomic structures make surgical exploration and complete resection a therapeutic challenge, which precludes local control in the majority of cases. Additionally, limited or delayed radiotherapy (RT) in young children decreases treatment outcomes, and dissemination is increased via the cerebrospinal fluid (CSF) [2,8]. The presence of intracranial extension and cranial nerve palsies at diagnosis has been shown to herald a poor prognosis with the risk of developing local and distant recurrence [9,10].

Notwithstanding, results of a pooled analysis from North American and European cooperative groups [3] reported a great variation in survival for children with PM-RMS, which is not uniformly poor. Nonetheless, despite the improved outcome seen in high-income countries (HICs)—with five-year overall survival (OS) of over 60.0–70.0% [3], the treatment of PM-RMS remains exceptionally challenging in low-income countries (LICs) with inferior survival of 10%−45% [5,11,12]. However, there is a paucity of data on prognostic factors impacting outcomes in resource-limited settings such as Uganda. This study, therefore, aimed to determine the clinical profile and predictors of outcomes of PM-RMS in children and adolescents treated at a single tertiary cancer treatment facility in Uganda.

## Methods

### Study design and setting

This study was a retrospective review of records of children and adolescents under 18 years diagnosed and treated for PM-RMS at the Uganda Cancer Institute (UCI) between January 2016 and December 2020. The UCI is Uganda’s only national reference cancer treatment center, treating nearly 80% of the children with cancer in the country with nearly 400–500 new diagnoses of childhood cancer annually, making it a representative site in the country for conducting the study.

### Study population and eligibility

**Fig 1:**
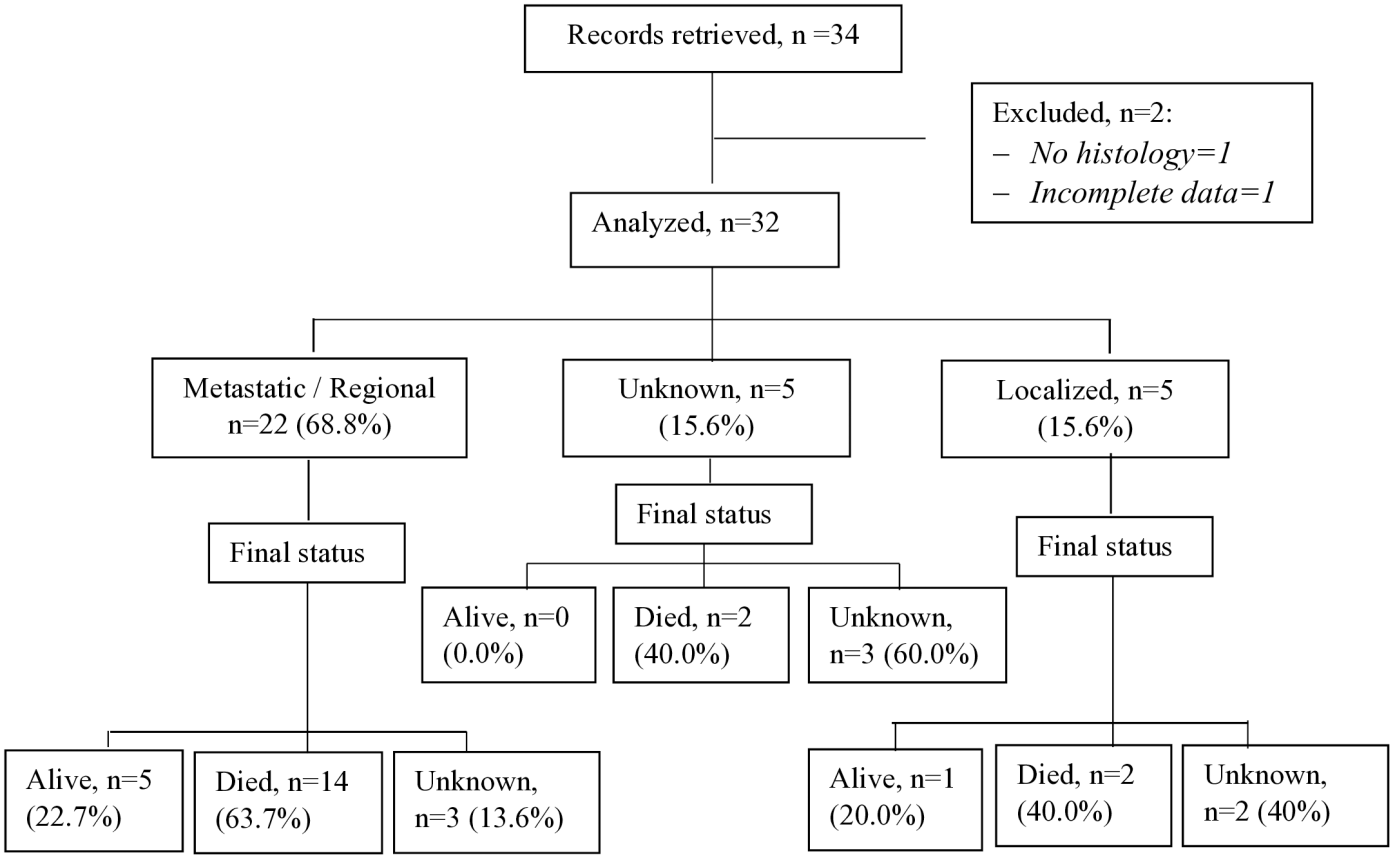
Study flow diagram.

The study included children and adolescents aged less than 18 years treated for PM-RMS at the UCI between January 2016 and December 2020. We excluded patients who had an uncertain or inconclusive diagnosis, incomplete medical records that lacked clinical details, or an alternative diagnosis upon histology review (see Fig 1).

### Study procedure and data extraction

We accessed the records and collected data for this study from November 05, 2022, to February 26, 2023. We consecutively retrieved and reviewed all accessible patient records within the study period, and all eligible participants were enrolled. The data collected encompassed demographic information, including age and sex/gender, the duration of symptoms, tumor characteristics, the extent of disease, patient management, and details regarding the clinical course and outcomes.

### Rhabdomyosarcoma diagnosis and treatment

The diagnosis of RMS was made on the basis of clinical presentations and radiological findings. Histological confirmation of the diagnosis was performed by experienced pathologists at the institute in accordance with criteria defined by the World Health Organization (WHO) classification [13]. However, molecular and cytogenetic studies were not available at the UCI. The primary tumor site and local extent of the tumor at diagnosis were assessed by computed tomography (CT). The initial staging workup included a CT scan of the chest or chest X-ray and bone marrow biopsies and trephine examination. Cerebrospinal fluid analysis was not routine. Primary surgical resection was attempted if gross tumor resection was deemed feasible; otherwise, only a biopsy was performed for diagnosis, and neoadjuvant chemotherapy was initiated. Pre-surgical clinical staging was assigned according to the Intergroup Rhabdomyosarcoma Study Group (IRSG) pre-surgical staging classification [14]. Clinical grouping was assigned according to the IRSG postsurgical grouping classification.

The treatment of PM-RMS in children at the study institution uses a locally adapted protocol based on the Children’s Oncology Group (COG) protocol. Patients received a chemotherapy combination with vincristine, doxorubicin, and cyclophosphamide (VDC) alternating with vincristine, actinomycin D, and cyclophosphamide (VAC) every 3 weeks (S1 Fig), or VDC alternating with ifosfamide, vincristine, and actinomycin D (IVA) every 3 weeks, depending on risk stratification (S2 Fig). Patients also received uroprotection with mesna during cyclophosphamide and ifosfamide treatment to prevent hemorrhagic cystitis. Granulocyte colony-stimulating factor (G-CSF) support was used in the event of bone marrow suppression resulting in neutropenia of less than 500 cells/µL. Local control consisted of surgery, radiation therapy, or both. Surgical excision of the primary tumor was attempted where feasible after 4 cycles of neoadjuvant chemotherapy, followed by radiotherapy to the primary site. Where surgical excision was not feasible, radiotherapy followed neoadjuvant chemotherapy.

### Clinical and outcome definitions

Localized disease was defined as RMS confined to the site or organ of origin that hasn’t spread to other parts of the body.

Regional nodal involvement was defined as RMS that has spread beyond the site or organ of origin to involve regional lymph nodes.

Metastatic disease was defined as RMS that has spread beyond the site or organ of origin to involve distant organs such as the lungs, bone, bone marrow, and liver, including lymph node metastasis other than regional nodal metastasis.

Overall survival (OS) was defined as the time duration from the date of diagnosis to death from any cause or to the date the patient was last known to be alive.

Event-free survival (EFS) was defined as the time duration from the date of diagnosis to the first event (disease progression, relapse, or death from any cause, whichever came first).

Abandonment was defined in line with the International Society of Pediatric Oncology (SIOP) recommendations as the failure to initiate or complete curative treatment during four or more consecutive weeks (except in situations when such treatment is contraindicated for medical reasons—e.g., the patient is too ill) [15,16].

### Statistical analysis

Data were analyzed using the Statistical Package for Social Sciences (SPSS) software package (SPSS for Windows, Version 27.0. Chicago, SPSS Inc.). Descriptive statistics were summarized as proportions for categorical variables, while continuous variables were summarized as means (standard deviation) if normally distributed or medians (interquartile range) if non-normally distributed. Survival analysis was estimated using the Kaplan-Meier method and compared using the log-rank test [17]. Patients alive at the end of the period under consideration for survival analysis or at the last follow-up date were censored. Likewise, patients with unknown outcomes—such as those lost to follow-up or who had not experienced the event of interest by the end of the study period—were right-censored at their last known follow-up date, consistent with standard survival analysis methodology [17]. This means that their data contributed to the risk up to the point of their last recorded follow-up, but not beyond. The effect of covariates on survival was estimated using Cox proportional hazards analysis [18]. The variables found to be statistically significant on univariate analysis were included in the multivariate model. Hazard ratios (aHR) were generated with the associated 95% confidence intervals (CI). A two-sided p-value <0.05 was considered for statistical significance. Due to the limited sample size (n = 32), we acknowledge that multivariable and subgroup analyses may be underpowered and some associations may be unstable. The study was not designed to detect small effect sizes or perform extensive subgroup analyses with statistical confidence.

### Ethics approval and consent to participate

All methods were carried out in accordance with relevant guidelines and regulations, and the study was conducted in accordance with the Declaration of Helsinki. The study was approved by the Uganda Cancer Institute Research and Ethics Committee (UCIREC-2022–45). Written informed consent and assent were accordingly waived by the research and ethics committee. While the records had identifiers like participants’ names, the study used codes in place of these identifiers to maintain confidentiality during data collection and throughout the study.

## Results

### Description of the study participants

During the study period, data for 32 children and adolescents treated for parameningeal rhabdomyosarcoma were analyzed (Fig 1).

The median age at diagnosis was 4.8 years (range 1–15 years), and the majority of the patients were aged below 10 years. The infratemporal region (base of skull) accounted for a quarter (25.0%) of the PM-RMS sites, followed by the middle ear (n = 7, 21.8%), nasopharynx (n = 6, 18.8%), paranasal sinus (n = 6, 18.8%), and nasal cavity (n = 5, 15.6%). Twenty-six (81.2%) of the tumors were locally invasive (T2) —i.e., tumors extending and/or infiltrating surrounding tissues; six (18.8%) of the patients had intracranial extension of the tumor. Histologically, 15 (46.9%) were embryonal, and seven (21.8%) were alveolar, while 10 (31.3%) were not typed. In general, five (15.6%) of the tumors were confirmed to be localized, 11 (34.4%) were associated with regional nodal involvement, two (6.3%) had distant metastasis to the lungs and liver, and 6 (18.8%) had extension into the intracranium. The majority, 17 (53.2%), were classified as intermediate risk, while seven (21.8%) were high-risk (Table 1).

**Table 1:** Demographic and disease characteristics (n = 32).

Variable	N	%
Age (years)		
< 10	25	78.1
≥ 10	7	21.9
Sex		
Female	9	28.1
Male	23	71.9
Tumor location		
Nasal cavity	5	15.6
Nasopharynx	6	18.8
Paranasal sinus	6	18.8
Middle ear	7	21.8
Base of skull	8	25.0
Tumor invasiveness		
T1	4	12.5
T2	26	81.2
Tx	2	6.3
Intracranial extension		
Yes	6	18.8
No	26	81.2
Tumor histology		
Embryonal	15	46.9
Alveolar	7	21.8
RMS NOS	10	31.3
IRS group		
II	1	3.1
III	29	90.6
IV	2	6.3
Regional lymph mode involvement		
N1	11	34.4
N0	18	56.2
Nx	3	9.4
Overall Extent of disease		
Localized	5	15.6
Regional	11	34.4
Distant metastasis	2	6.3
Unknown	14	15.6
Risk stratification		
Low risk	3	9.4
Intermediate risk	17	53.2
High risk	7	21.8
Unknown	5	15.6

*T1 Tumor confined to anatomic site of origin (non-invasive); T2 Tumor extension and/or infiltration of surrounding tissues (invasive); Tx, Nx – not assessed; RMS NOS, Rhabdomyosarcoma not otherwise specified*.

### Common presenting symptoms of PM-RMS

All 32 patients had swelling as a presenting symptom, with pain as the second most common symptom (n = 16, 50.0%). Constitutional symptoms were present in half (50.0%) of the patients. The majority (n = 15, 46.9%) had symptoms for a duration of three to six months prior to presenting at the cancer treatment center, while eight patients (25.0%) had symptoms that had lasted more than six months (see Table 2).

**Table 2:** Common presenting symptoms in children and adolescent with PM-RMS (n = 32).

Variable	n	%
Clinical symptoms*		
Swelling	32	100
Pain	16	50.0
Headache	4	12.5
Loss of appetite	1	3.1
Hearing loss	6	18.8
Proptosis	1	3.1
Difficulty chewing	4	12.5
Constitutional symptoms		
Yes	16	50.0
No	16	50.0
Specific constitutional symptoms^¥^		
Fever	13	40.6
Weight loss	10	31.3
Drenching night sweats	10	31.3
Duration of symptoms (months)		
<3	9	28.1
3-6	15	46.9
>6	8	25.0

*Multiple symptoms applicable; ^*¥*^*proportion of those who had B symptoms*.

### Treatment characteristics and clinical outcomes

Less than half (n = 13; 40.6%) of the patients were discussed in the multidisciplinary team meetings (MDM) in the course of their management. Almost all the patients (29, 90.6%) received chemotherapy, mainly VDC/VAC (18, 62.1%). Fifteen (42.9%) patients received local control, of which 14 received radiotherapy alone, and only one patient had surgical resection, while none received both irradiation and surgery. Four of 15 patients (26.7%) who abandoned treatment had received radiotherapy. Six (18.8%) of the patients were alive at the time of the study, 18 (56.2%) had died, and the status of eight (25.0%) of the patients could not be ascertained (Table 3).

**Table 3:** Treatment characteristics and clinical outcomes (n = 32).

Variable	n	%
Discussed in MDM		
Yes	13	40.6
No	19	59.4
Chemotherapy		
Yes	29	90.6
No	3	9.4
Chemotherapy regimen (n = 29)		
VDC/VAC	18	62.1
VDC/IVA	4	13.8
Both*	7	24.1
Definitive surgery		
Yes	1	3.1
No	31	96.9
Radiotherapy		
Yes	14	43.8
No	18	56.2
Final outcome		
Alive	6	18.8
Died	18	56.2
Unknown	8	25.0

MDM, Multidisciplinary meeting *VDC/VAC then switched to VDC/IVA due to progressive disease, VDC (vincristine, doxorubicin, cyclophosphamide), VAC, (vincristine, actinomycin D, cyclophosphamide); IVA (Ifosfamide, vincristine, actinomycin D).

### Events during management

Fourteen patients experienced disease progression during the course of their management, 12 (85.7%) of whom died. There were no documented relapses, but up to 15 (46.9%) of the children with PM-RMS abandoned treatment, nine (60.0%) of whom were confirmed to have died, while the status of the other six remained unknown.

### Survival outcomes

**Fig 2:**
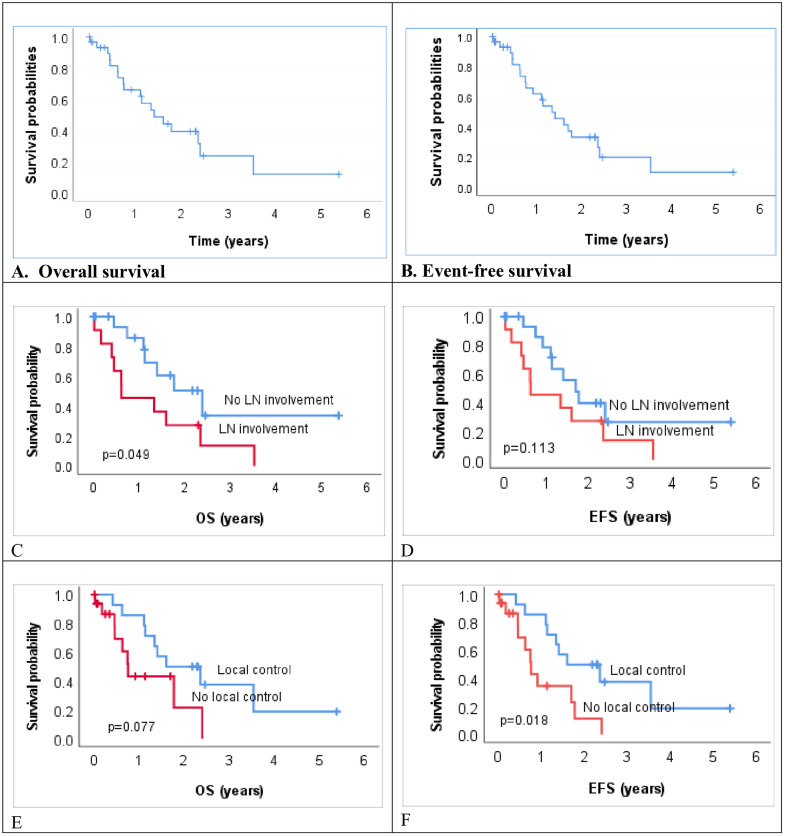
Survival curves for overall and event-free survival in children and adolescents with PM-RMS. (A) Overall survival (OS). (B) Event-free survival (EFS). (C) OS by regional lymph node (LN) involvement. (D) EFS by regional lymph node (LN) involvement. (E) OS by local control status. (F) EFS by local control status.

The median overall survival (OS) was 1.4 years (95% CI 0.7–2.1), while the one-, three-, and five-year probabilities of overall survival were 65%, 23%, and 12%, respectively (Fig 2A). The median event-free survival (EFS) was 1.3 years (95% CI 0.8–1.9), while the one-, three-, and five-year probabilities of EFS were 62%, 20%, and 10%, respectively (Fig 2B). The absence of nodal involvement was associated with significantly improved overall survival (OS) compared to cases with nodal involvement (p = 0.049; Fig 2C). Event-free survival (EFS) was also higher in children without nodal involvement, although the difference did not reach statistical significance (p = 0.113; Fig 2D). Local control was associated with improved OS (p = 0.077; Fig 2E) and a statistically significant improvement in EFS (p = 0.019; Fig 2F).

### Determinants of survival among children with PM-RMS

In bivariate analysis, lack of multidisciplinary management was associated with significantly inferior OS (HR 4.00; 95% CI 139–11.48; p = 0.010) and EFS (HR 2.95; 95% CI 1.15–7.58; p = 0.025). In addition, EFS was significantly inferior for patients who did not receive radiation therapy for local control (HR 2.95; 95% CI 1.16–7.47; p = 0.023) (Table 4).

**Table 4:** Bivariate Cox proportional hazards model for Overall survival (n = 32).

Model	OS^¶^ (%)	Overall survival			EFS^¶^ (%)	Event-free survival		
		HR	95% CI	p value		HR	95% CI	p value
Sex								
Female	42	Ref			33	Ref		
Male	38	1.17	0.38-3.61	0.782	32	1.00	0.36-2.78	0.999
Age (years)								
<10	43	Ref			35	Ref		
≥ 10	28	1.82	0.67-4.96	0.245	28	1.53	0.57-4.06	0.396
Duration of symptoms¥								
< 6 months	32	Ref			30	Ref		
≥ 6 months	55	1.31	0.46-3.73	0.612	41	1.20	0.45-3.15	0.718
Local tumor extension								
T1	50	Ref			50	Ref		
T2	39	1.16	0.33-4.07	0.822	31	1.34	0.39-4.64	0.644
Nodal involvement								
N0	50	Ref			40	Ref		
N1	28	2.57	0.97-6.80	0.057	28	2.05	0.83-5.08	0.120
Intracranial extension								
No	44	Ref			36	Ref		
Yes	21	1.79	0.62-5.17	0.280	20	1.63	0.57-4.59	0.360
Stage								
1/2	100	Ref			67	1		
3/4	33	2.60	0.57-11.77	0.215	30	1.84	0.52-6.51	0.346
Risk strata								
LR/	43	Ref			40	Ref		
IR/HR	43	1.46	0.49-4.36	0.501	28	1.58	0.57-4.35	0.380
Histology								
Embryonal	39	Ref			33	Ref		
Alveolar	00	1.66	0.52-5.26	0.388	00	1.42	.46-4.36	0.541
MDM								
Yes	55	Ref			43	Ref		
No	24	4.00	1.39-11.48	**0.010**	24	2.95	1.15-7.58	**0.025**
Irradiated								
Yes	50	Ref			50	Ref		
No	22	2.36	0.89-6.26	0.085	12	2.95	1.16-7.47	**0.023**
Treatment abandonment								
No	46	Ref			35	Ref		
Yes	30	1.91	0.73-4.97	0.186	30	2.46	0.62-3.81	0.350

*¶Two-year survivals; ¥denotes length of time that a patient had symptoms before presentation; T1. Not locally invasive; T2, Locally invasive; N0, No lymph node involvement; N1, lymph node involvement; LR, Low-risk; IR, Intermediate-risk; HR, High-risk; MDM, Multidisciplinary management*.

In multivariate analysis, two factors that independently predicted survival among children with PM-RMS were regional nodal involvement and the lack of radiation therapy. OS and EFS were significantly inferior for children with nodal involvement, with adjusted hazard ratios (aHR) of 4.95 (95% CI 1.53–15.98, p = 0.008) and 4.61 (95% CI 1.47–14.40, p = 0.009), respectively. The absence of radiation therapy was also significantly associated with poorer overall survival (OS) and event-free survival (EFS), with adjusted hazard ratios (aHR) of 4.79 (95% CI 1.40–16.36, p = 0.012) for OS and 6.07 (95% CI 1.85–19.98, p = 0.003) for EFS, respectively (Table 5).

**Table 5:** Multivariate Cox proportional hazards model for Overall survival (n = 32).

Model	Overall survival			Event-free survival		
	aHR	95% CI	p value	aHR	95% CI	p value
Nodal involvement						
N0	Ref			Ref		
N1	4.95	1.53-15.98	**0.008**	4.61	1.47-14.40	**0.009**
MDM						
Yes	Ref			Ref		
No	2.19	0.68-7.04	0.190	1.55	0.54-4.48	0.420
Irradiated						
Yes	Ref			Ref		
No	4.79	1.40-16.36	**0.012**	6.07	1.85-19.98	**0.003**
Treatment abandonment						
No	Ref			–	–	–
Yes	1.54	0.45-5.23	0.490			

*MDM, Multidisciplinary Management*.

## Discussion

The treatment and outcomes of PM-RMS remain a significant challenge, especially in the resource-limited settings of LMICs. The current study demonstrated inferior survival among children with PM-RMS compared to those observed in HICs, as well as other LMIC settings. The prognosis of PM-RMS was significantly related to the regional nodal involvement and local control, particularly radiation therapy.

### Common clinical characteristics of PM-RMS

Our study demonstrated a generally younger age distribution for children with PM-RMS, with a median age of 4.8 years. The age distribution in our cohort differs from reports by Kraus et al. [19] and Douglas et al. [20], where the median ages were much higher at 7.7 and 8.5 years (range 1.5–19 years), respectively. The younger age in our cohort compared to that reported in the other studies may partly be attributed to the differences in the inclusion criteria, with the maximum age in the current study being 17 years while the other studies included patients up to 19 years. Nonetheless, our finding appears coherent with the age demographic in RMS in general, a disease considered the most common soft tissue sarcoma of early childhood [21]. We found a male predominance among children with PM-RMS, which is consistent with reports by Parambil et al. in India [2], Douglas et al. in the USA [20], and other authors [22,23].

The most common tumor site in our study was the infratemporal region (base of skull), which mirrors reports by Resham et al. in Pakistan [12] but differs from other reports where nasopharynx, maxillary, and parapharyngeal sites were the most commonly involved [20,22]. In about a quarter of the patients, more than one of the parameningeal sites was involved, and most of the patients presented with a locally advanced tumor. This finding is consistent with that reported by Rahman et al. in Egypt [22] and is of great clinical significance since this impacts the possibility of both surgical resection and radiation therapy as modalities for local control management.

Embryonal histology predominated in our patient cohort, corroborating results reported in literature [12,24]—but with a lower proportion than that observed in most reports. For instance, in a study by Rahman et al. in Egypt, the embryonal pathological subtype accounted for up to 73.8% of the cases [22], with an even much higher rate (88%) observed by Resham et al. in Pakistan, where only 9% of the patients had alveolar histology [12]. The observed contrast in the rates of embryonal histology, a favorable histological subtype of RMS, between our results and those of other reports could possibly relate to the limitations of reliance on morphological histopathological assessment in the absence of molecular studies—resulting in a significant proportion (31%) of patients with RMS not otherwise specified in our study. The lack of molecular subtyping (e.g., PAX3/7-FOXO1 fusion testing) —increasingly recognized as a key prognostic factor [25] —limited our ability to definitively classify rhabdomyosarcoma subtypes. This could have introduced potential misclassification, particularly between embryonal and alveolar subtypes, as has been previously documented in the literature [26,27].

The majority of the patients in our cohort were in IRS group III and stage 3 at diagnosis, which is similar to that reported by Resham et al. in Pakistan [12]. The predominance of stage 3 disease as found in our patient cohort has also been reported in other studies [22]. However, while these studies also report an appreciable proportion of patients in the lower stages—23.8% [22] and 46% [12] of patients in stage 2—this was an uncommon finding among the patient population in the current cohort. The finding of a high rate of advanced-stage disease in PM-RMS has been attributed to diseases in these sites being less visible than other superficial head and neck sites [28] and the tendency to leptomeningeal progression [29]. Our finding is consistent with that from other African regions, as exemplified by reports from South Africa and Egypt, where up to 91% and 76.1%, respectively, of pediatric RMS patients presented with advanced (stage III or IV) disease [5,22].

Only one child with PM-RMS was operated on in our cohort—which falls short of the 60% rate reported in a Chinese study [30]. That notwithstanding, surgery in PM-RMS is reported to be generally low [31] —consequent to the special anatomical site and infiltrative nature of PM-RMS. These make surgery very difficult without impinging on important structures and functions. For instance, in one study, maxillofacial resection was associated with a 31.3% mortality and 48.3% recurrence rate [32]. Chemo-radiation therefore remains the standard treatment for PM-RMS, with surgery often limited to biopsy or salvage therapy for recurrent disease [33]. However, where feasible, surgery has been associated with a significantly higher survival rate [31]. The low rate of surgical resection in the current study could be attributed to limited institutional surgical capacity at the time. The fact that many more children received radiotherapy in our setting, with an improved outcome, is reassuring and can be a benchmark for improved surgical capacity.

### Survival outcomes and prognostic factors for survival

Despite the availability of multimodal treatment protocols, we observed very low three- and five-year OS and EFS rates for children with PM-RMS in the current study. These observed survival rates are much inferior to those reported in high-income countries [3,30], as well as other LMICs [22]. Our finding contrasts that from an Egyptian study where the three-year OS and EFS rates were higher (58.4% and 48%, respectively) [22], but mirrors that reported by Resham et al. in Pakistan, where the three-year OS and EFS were low, at 37% and 23%, respectively [12]. The observed rates in the current study could partly be attributed to the high (46.9%) treatment abandonment rate among our patient cohort, 60% of whom died—a challenge inherent among pediatric cancer patients in most LMICs, especially the African region—as also reported in Zambia (46%) and Kenya (54%) in a multicenter sub-Saharan African study [34]. The challenge of abandonment, and thus treatment interruption, is concerning since both dose intensity and cumulative dose of cyclophosphamide have been shown to affect outcomes in PM-RMS, with significantly better local control rates and a trend towards better EFS when the desired cumulative dose of cyclophosphamide is used [35].

Survival rates among our patient cohort were significantly lower for patients with regional lymph node involvement, which remained an important predictor of survival (both OS and EFS) in the current study. The prognostic significance of nodal involvement in RMS in general has long been documented [36,37]. In this study, for example, the lack of surgical resection in general and of lymph node resection in particular could be a significant factor. As shown by Machavoine et al. [38], lymph node surgery in PM-RMS may improve the survival of these patients. This is an important point for discussion during multidisciplinary team meetings since it is an actionable aspect of treatment with prognostic significance. However, this study included a relatively small cohort (n = 32) which could have inherently limited the power of subgroup comparisons and the precision of hazard ratio estimates as reflected by the wide confidence intervals, and calls for cautious interpretation. These constraints highlight the need for larger studies to confirm the observed associations.

Survival rates were significantly better for patients who received local control, largely radiation therapy (RT), a finding similar to that reported by Peng et al. in China, where the OS and EFS were significantly higher among patients who received RT than the non-RT group (3-year OS: 85.6% vs. 0% and 3-year EFS: 64.0% vs. 0%) [39]. These findings confirm the importance of RT as an effective means of local control in PM-RMS [3,40], without which a cure is believed to be unlikely [41]. While concerns about the acute toxicity and late effects of RT in young children are true, omission or delay of RT significantly increases the local recurrence rate even in infants and children younger than 3 years [9]. Progressive RT technologies such as intensity-modulated radiotherapy (IMRT) and proton radiotherapy (PT) are well-tolerated with mild-moderate toxicity and have been used in HICs [42,43], but these modalities are unavailable in resource-limited settings. Less than half of our patient cohort received RT—typically 54 Gy in 28 fractions—translating to 1.8 Gy per session, delivered either using 2D conventional RT (before 2021) or 3D conformal RT (3D-CRT) (from 2021 onwards). This gap could be attributed to barriers such as cost of RT, the long lead time to RT due to patient load, and often missed opportunities for RT because of the need for sedation for children.

The prognostic significance of the primary site of PM-RMS has shown inconsistent results. A pooled analysis from North American and European cooperative groups found that site was significantly correlated with outcome, with infratemporal and pterygopalatine fossa and paranasal sinus sites portending the worst prognosis, while other PM sites showed better outcomes [3]. Contrastingly, the current study did not demonstrate a statistically significant difference in survival outcomes on the basis of the primary site. This finding correlates with that reported by Parambil et al. and Duan et al., where the site of primary had no prognostic significance [2,30]. The small sample sizes in the current and the other studies could probably explain the observed results. In addition, the prognostic effect of the site seems to be influenced by radiotherapy, without which the tumor site has no significant prognostic effect [3]. Thus, a larger prospective study would be necessary to validate the prognostic significance of the sites of primary tumor in PM-RMS.

The major limitations of the current study relate to those inherent to a retrospective design as well as the relatively small sample size. The small sample size represents a significant limitation of this study. It restricts the statistical power of subgroup and multivariate analyses, increases the risk of Type II error, and results in imprecise hazard ratio estimates, as evidenced by wide confidence intervals. These limitations reduce the generalizability of our findings and highlight the need for larger studies to confirm the observed associations. Nonetheless, this cohort reflects real-world clinical experience in a resource-constrained setting, where data are limited and often difficult to collect. The limitations in the monocentric sample source also warrant consideration and may have affected the external validity of our findings and hence their generalizability. The lack of molecular and cytogenetic studies also precluded evaluation of other prognostic factors, especially the molecular status, which was unavailable in the study context. Nonetheless, being a rare subset of RMS, the current study underscores valuable aspects of PM-RMS, which could be the basis for further prospective studies in a larger multicenter cohort.

### Conclusion and recommendations

The findings from this study suggest that resource context may significantly influence treatment outcomes for childhood cancers such as PM-RMS. The relatively poorer outcomes observed in this cohort drawn from a resource-limited setting highlight potential systematic challenges that warrant further investigation. Efforts to improve local control measures—including access to surgery and radiation therapy—alongside initiatives to raise community awareness for early detection and reduce treatment abandonment, may contribute to better outcomes. Our findings should serve as a basis for larger prospective studies to inform targeted interventions.

## Supporting information

S1 Fig Treatment protocol for All group 3: Including Parameningeal stage 3 and Extremity stage 3, Favorable histology only. (TIF)

S2 Fig Treatment protocol for Group 4: All Metastatic Tumors, 2. All Unfavorable Histology, Group 3: Parameningeal Satge 3 and Extremity Stage 3. (TIF)

## Abbreviations

CSF: Cerebrospinal fluid
CT: Computed tomography
GCSF: granulocytic colony stimulating factor
HIC: High-income country
IMRT: Intensity-modulated radiotherapy
IRSG: Intergroup rhabdomyosarcoma study group
IVA: Ifosfamide plus Vincristine plus Adrimycin
LDH: serum lactate dehydrogenase
LIC: Low-income countries
MDM: Multidisciplinary management
OS: Overall survival
PM-RMS: Parameningeal; rhabdomyosarcoma
PT: Proton radiotherapy
RMS: Rhabdomyosarcoma
RMS-NOS: Rhabdomyosarcoma not otherwise specified
RT: Radiotherapy
UCI: Uganda Cancer Institute
VAC: Vincristine plus Adriamycin plus Cyclophosphamide
VDC: Vincristine plus Doxorubicin plus Cyclophosphamide
WHO: World Health Organization

## References

[1] Choi PJ, Iwanaga J, Tubbs RS, Yilmaz E. Surgical interventions for advanced parameningeal rhabdomyosarcoma of children and adolescents. Cureus. 2018;10(1):e2045. doi: 10.7759/cureus.2045 29541566 PMC5844646

[2] Parambil BC, Gollamudi V, Prasad M, Patel V, Laskar S, Khanna N, et al. Parameningeal rhabdomyosarcoma- clinical prole, outcomes and prognostic factors in children treated at a single center over a decade. Authorea. 2024. doi: 10.22541/au.171663389.195078793/v171663381 40168045

[3] Merks JHM, De Salvo GL, Bergeron C, Bisogno G, De Paoli A, Ferrari A, et al. Parameningeal rhabdomyosarcoma in pediatric age: results of a pooled analysis from North American and European cooperative groups. Ann Oncol. 2014;25(1):231–6. doi: 10.1093/annonc/mdt426 24356633 PMC3868324

[4] Papyan R, Tamamyan G, Danielyan S, Tananyan A, Muradyan A, Saab R. Identifying barriers to treatment of childhood rhabdomyosarcoma in resource-limited settings: a literature review. Pediatr Blood Cancer. 2019;66(7):e27708. doi: 10.1002/pbc.27708 30907501

[5] Van Der Schyff A, Stefan DC. Clinical characteristics and outcome of rhabdomyosarcoma in South African children. Afr J Haematol Oncol. 2010;1(2):40–7.

[6] Skapek SX, Ferrari A, Gupta AA, Lupo PJ, Butler E, Shipley J, et al. Rhabdomyosarcoma. Nat Rev. 2019;5(1). 10.1038/s41572-018-0051-2 PMC7456566 30617281

[7] Radzikowska J, Kukwa W, Kukwa A, Czarnecka A, Krzeski A. Rhabdomyosarcoma of the head and neck in children. Contemp Oncol (Pozn). 2015;19(2):98–107. doi: 10.5114/wo.2015.49158 26034386 PMC4444444

[8] Liuzzi JF, Da Cunha M, Salas D, Siso S, Garriga E. Soft-tissue sarcomas in the head and neck: 25 years of experience. Ecancermedicalscience. 2017;11:740. doi: 10.3332/ecancer.2017.740 28626490 PMC5464559

[9] Michalski JM, Meza J, Breneman JC, Wolden SL, Laurie F, Jodoin M, et al. Influence of radiation therapy parameters on outcome in children treated with radiation therapy for localized parameningeal rhabdomyosarcoma in Intergroup Rhabdomyosarcoma Study Group trials II through IV. Int J Radiat Oncol Biol Phys. 2004;59(4):1027–38. doi: 10.1016/j.ijrobp.2004.02.064 15234036

[10] Turner JH, Richmon JD. Head and neck rhabdomyosarcoma: a critical analysis of population-based incidence and survival data. Otolaryngol Head Neck Surg. 2011;145(6):967–73. doi: 10.1177/0194599811417063 21873599

[11] Bhurgri Y, Bhurgri A, Puri R. Rhabdomyosarcoma in Karachi 1998–2002. Asian Pac J Cancer Prev. 2004;5:284–90. 15373708

[12] Resham S Dr, Raza MR Dr, Qureshi BM, Rizvi A, Ashraf MS Dr, Altaf S Dr. Prognostic factors and their influence on therapeutic outcomes in children and adolescents with parameningeal rhabdomyosarcoma: a multicenter study from Pakistan. Pediatr Hematol Oncol J. 2021;6(1):6–11. doi: 10.1016/j.phoj.2020.02.002

[13] Stefan DC. Patterns of distribution of childhood cancer in Africa. J Trop Pediatr. 2015;61(3):165–73. doi: 10.1093/tropej/fmv005 25724211

[14] Crist WM, Anderson JR, Meza JL, Fryer C, Raney RB, Ruymann FB, et al. Intergroup rhabdomyosarcoma study-IV: results for patients with nonmetastatic disease. J Clin Oncol. 2001;19(12):3091–102. doi: 10.1200/JCO.2001.19.12.3091 11408506

[15] Mostert S, Arora RS, Arreola M, Bagai P, Friedrich P, Gupta S, et al. Abandonment of treatment for childhood cancer: position statement of a SIOP PODC Working Group. Lancet Oncol. 2011;12(8):719–20. doi: 10.1016/S1470-2045(11)70128-0 21719348

[16] Njuguna F, Mostert S, Slot A, Langat S, Skiles J, Sitaresmi MN, et al. Abandonment of childhood cancer treatment in Western Kenya. Arch Dis Child. 2014;99(7):609–14. doi: 10.1136/archdischild-2013-305052 24681695

[17] Kaplan EL, Meier P. Nonparametric estimation from incomplete observations. J Am Stat Assoc. 1958;53(282):457–81. doi: 10.1080/01621459.1958.10501452

[18] Cox DR. Regression models and life tables. J R Stat Soc B. 1972;34:187–220.

[19] Kraus DH, Saenz NC, Gollamudi S, Heller G, Moustakis M, Gardiner S, et al. Pediatric rhabdomyosarcoma of the head and neck. Am J Surg. 1997;174(5):556–60. doi: 10.1016/s0002-9610(97)00171-2 9374237

[20] Douglas JG, Arndt CAS, Hawkins DS. Delayed radiotherapy following dose intensive chemotherapy for parameningeal rhabdomyosarcoma (PM-RMS) of childhood. Eur J Cancer. 2007;43(6):1045–50. doi: 10.1016/j.ejca.2007.01.033 17368885

[21] Gradoni P, Giordano D, Oretti G, Fantoni M, Barone A, La Cava S, et al. Clinical outcomes of rhabdomyosarcoma and Ewing’s sarcoma of the head and neck in children. Auris Nasus Larynx. 2011;38(4):480–6. doi: 10.1016/j.anl.2010.12.004 21227608

[22] Rahman HA, Sedky M, Mohsen I, Taha H, Loaye I, Zaghloul MS, et al. Outcome of pediatric parameningeal rhabdomyosarcoma. The Children Cancer Hospital, Egypt, experience. J Egypt Natl Canc Inst. 2013;25(2):79–86. doi: 10.1016/j.jnci.2013.01.002 23719406

[23] Andrade CR de, Takahama Junior A, Nishimoto IN, Kowalski LP, Lopes MA. Rhabdomyosarcoma of the head and neck: a clinicopathological and immunohistochemical analysis of 29 cases. Braz Dent J. 2010;21(1):68–73. doi: 10.1590/s0103-64402010000100011 20464324

[24] Meazza C, Ferrari A, Casanova M, Gandola L, Collini P, Massimino M, et al. Evolving treatment strategies for parameningeal rhabdomyosarcoma: the experience of the Istituto Nazionale Tumori of Milan. Head Neck. 2005;27(1):49–57. doi: 10.1002/hed.20117 15529318

[25] Rudzinski ER, Anderson JR, Chi Y-Y, Gastier-Foster JM, Astbury C, Barr FG, et al. Histology, fusion status, and outcome in metastatic rhabdomyosarcoma: a report from the Children’s Oncology Group. Pediatr Blood Cancer. 2017;64(12):10.1002/pbc.26645. doi: 10.1002/pbc.26645 28521080 PMC5647228

[26] Williamson D, Missiaglia E, de Reyniès A, Pierron G, Thuille B, Palenzuela G, et al. Fusion gene-negative alveolar rhabdomyosarcoma is clinically and molecularly indistinguishable from embryonal rhabdomyosarcoma. J Clin Oncol. 2010;28(13):2151–8. doi: 10.1200/JCO.2009.26.3814 20351326

[27] Parham DM, Ellison DA. Rhabdomyosarcomas in adults and children: an update. Arch Pathol Lab Med. 2006;130(10):1454–65. doi: 10.5858/2006-130-1454-RIAACA 17090187

[28] Wiener ES. Head and neck rhabdomyosarcoma. Semin Pediatr Surg. 1994;3(3):203–6. 7987636

[29] Raney RB Jr, Tefft M, Newton WA, Ragab AH, Lawrence W Jr, Gehan EA, et al. Improved prognosis with intensive treatment of children with cranial soft tissue sarcomas arising in non-orbital parameningeal sites. Cancer. 1987;59(1):147–55. doi: 10.1002/1097-0142(19870101)19870159:19870101<19870147::aid-cncr2820590129>2820590123.2820590120.co;2820590122-2820590128 3791141

[30] Duan C, He S, Ma X, Wang S, Jin M, Zhao W, et al. Clinical outcomes and prognostic factors of parameningeal rhabdomyosarcoma in children and adolescents: results of two consecutive protocols. Transl Pediatr. 2024;13(7):1086–96. doi: 10.21037/tp-24-41 39144439 PMC11320008

[31] Dombrowski ND, Wolter NE, Robson CD, Kawai K, Irace AL, Vargas SO, et al. Role of surgery in rhabdomyosarcoma of the head and neck in children. Laryngoscope. 2021;131(3):E984–92. doi: 10.1002/lary.28785 33107076

[32] Liu Z, Zhu F, Cao W, Sun J, Zhang C, He Y. Surgical treatment of pediatric rhabdomyosarcoma in the parameningeal-nonparameningeal region. J Craniomaxillofac Surg. 2020;48(1):75–82. doi: 10.1016/j.jcms.2019.12.002 31902716

[33] Casey DL, Mandeville H, Bradley JA, Ter Horst SAJ, Sheyn A, Timmermann B, et al. Local control of parameningeal rhabdomyosarcoma: an expert consensus guideline from the International Soft Tissue Sarcoma Consortium (INSTRuCT). Pediatr Blood Cancer. 2022;69(7):e29751. doi: 10.1002/pbc.29751 35484997

[34] Chagaluka G, Afungchwi GM, Landman L, Njuguna F, Hesseling P, Tchintseme F, et al. Treatment abandonment: a report from the collaborative African network for childhood cancer care and research-CANCaRe Africa. Pediatr Blood Cancer. 2021;68(12):e29367. doi: 10.1002/pbc.29367 34549506

[35] Casey DL, Wexler LH, Wolden SL. Worse outcomes for head and neck rhabdomyosarcoma secondary to reduced-dose cyclophosphamide. Int J Radiat Oncol Biol Phys. 2019;103(5):1151–7. doi: 10.1016/j.ijrobp.2018.11.049 30508617 PMC6441953

[36] Mandell L, Ghavimi F, LaQuaglia M, Exelby P. Prognostic significance of regional lymph node involvement in childhood extremity rhabdomyosarcoma. Med Pediatr Oncol. 1990;18(6):466–71. doi: 10.1002/mpo.2950180606 2233517

[37] Panda SP, Chinnaswamy G, Vora T, Prasad M, Bansal D, Kapoor G, et al. Diagnosis and management of rhabdomyosarcoma in children and adolescents: ICMR consensus document. Indian J Pediatr. 2017;84(5):393–402. doi: 10.1007/s12098-017-2315-3 28378141

[38] Machavoine R, Helfre S, Bernier V, Bolle S, Leseur J, Corradini N. Locoregional control and survival in children, adolescents, and young adults with localized head and neck alveolar rhabdomyosarcoma - the French experience. Front Pediatr. 2021;9:783754. doi: 10.3389/fped.2021.783754 35186818 PMC8855824

[39] Peng X, Xiong X, Li Y, Li C, Wang Z, Wu Y, et al. Local treatment of children suffering from parameningeal rhabdomyosarcoma: a retrospective single-center study from China. Cancer Control. 2024;31:10732748241240655. doi: 10.1177/10732748241240655 38514935 PMC10958813

[40] Affinita MC, Ferrari A, Milano GM, Scarzello G, De Leonardis F, Coccoli L, et al. Long-term results in children with head and neck rhabdomyosarcoma: a report from the Italian Soft Tissue Sarcoma Committee. Pediatr Blood Cancer. 2018;65(3):10.1002/pbc.26876. doi: 10.1002/pbc.26876 29115716

[41] Defachelles AS, Rey A, Oberlin O, Spooner D, Stevens MCG. Treatment of nonmetastatic cranial parameningeal rhabdomyosarcoma in children younger than 3 years old: results from international society of pediatric oncology studies MMT 89 and 95. J Clin Oncol. 2009;27(8):1310–5. doi: 10.1200/JCO.2008.1319.5701 19204197

[42] Weber DC, Ares C, Albertini F, Frei-Welte M, Niggli FK, Schneider R, et al. Pencil beam scanning proton therapy for pediatric parameningeal rhabdomyosarcomas: clinical outcome of patients treated at the Paul Scherrer Institute. Pediatr Blood Cancer. 2016;63(10):1731–6. doi: 10.1002/pbc.25864 26701148

[43] Lockney NA, Friedman DN, Wexler LH, Sklar CA, Casey DL, Wolden SL. Late toxicities of intensity-modulated radiation therapy for head and neck rhabdomyosarcoma. Pediatr Blood Cancer. 2016;63(9):1608–14. doi: 10.1002/pbc.26061 27195454 PMC4955714

