# Supplementary figures and images for: Clinicopathological profile and survival in children with parameningeal rhabdomyosarcoma in resource-limited settings: A single-center experience from Uganda
Source: PLoS One. 2025 Oct 9;20(10):e0334140. doi: 10.1371/journal.pone.0334140 (PMC12510527; doi:10.1371/journal.pone.0334140)

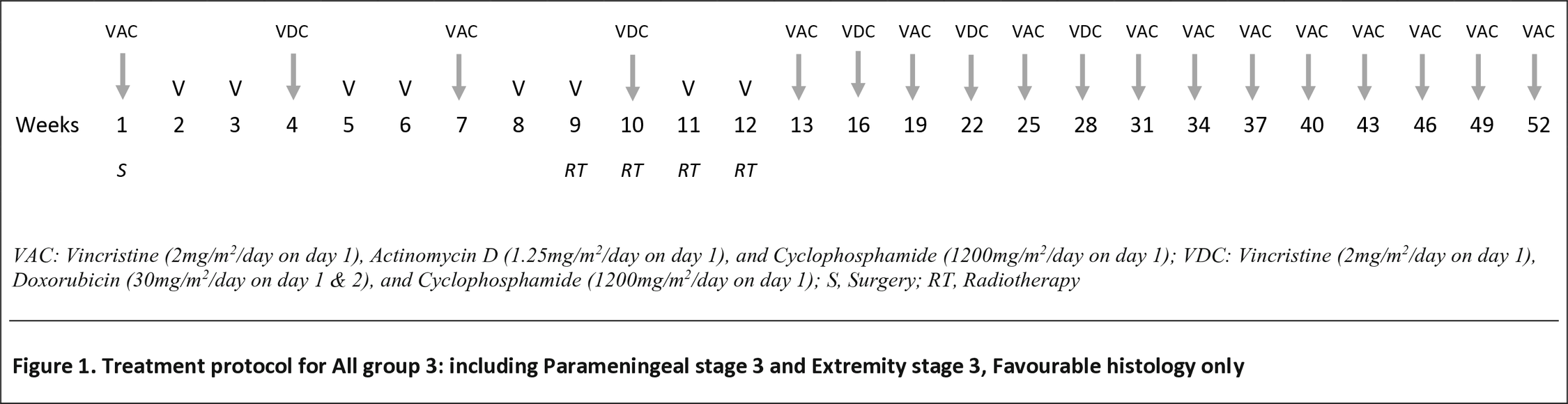

Supplement: S1 Fig — (TIF) [file pone.0334140.s001.tif]

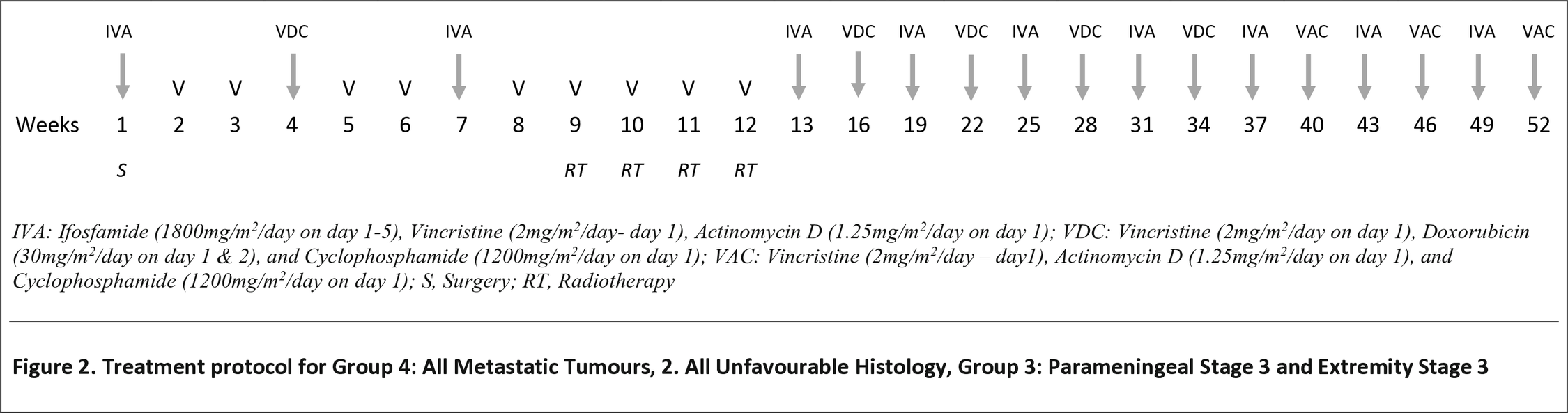

Supplement: S2 Fig — (TIF) [file pone.0334140.s002.tif]
